# Cloning, isolation, and characterization of novel chitinase-producing bacterial strain UM01 (*Myxococcus fulvus*)

**DOI:** 10.1186/s43141-020-00059-1

**Published:** 2020-08-31

**Authors:** Umar Shahbaz, Xiaobin Yu

**Affiliations:** The Key Laboratory of Carbohydrate Chemistry & Biotechnology, Ministry of Education, School of Biotechnology, Jiangnan University, 1800 Lihu Road, Wuxi, 214122 China

**Keywords:** *Myxococcus fulvus*, Gene cloning, Chitinase

## Abstract

**Background:**

Chitin is an important biopolymer next to cellulose, extracted in the present study. The exoskeleton of marine bycatch brachyuran crabs, namely Calappa lophos, Dromia dehaani, Dorippe facchino and also from stomatopod Squilla spp. were used to extract chitin through fermentation methods by employing two bacterial strains such as Pseudomonas aeruginosa, Serratia marcescens. The yield of chitin was 44.24%, 37.45%, 11.56% and 27.24% in C. lophos, D. dehaani, D. facchino and Squilla spp. respectively. FT-IR spectra of the produced chitin exhibit peaks which is more or less coherent to that of standard chitin which is further analysed by Scanning Electron Microscope. The quality of produced chitin was assessed through moisture, protein, ash and lipid content analysis ensured that chitin obtained from trash crustaceans are on par with that of standard chitin.

**Results:**

A total of 10 samples were collected from different areas of Jiangsu China for screening of chitinase-producing bacteria. Based on the clearance zone, two of the best samples were chosen for further study. 16S rRNA sequence analysis showed that this strain belongs to genus *Myxococcus* and species *Myxococcus fulvus*. Phylogenetic analysis was performed and it shows strain UM01 is a novel bacterial strain. UM01 isolate shows maximum chitinase production at 35 °C and 8 pH. Among all, these colloidal chitins were found to be the best for chitinase production. Three chitinase-producing genes were identified and sequenced by using degenerative plasmid. UMCda gene (chitin disaccharide deacetylase) was cloned into *E*. *coli DH5a* by using PET-28a vector, and antagonistic activity was examined against *T*. *reesei*.

**Conclusion:**

To our knowledge, this is the earliest study report to gene cloning and identification of the chitinase gene in *Myxococcus fulvus*. Chitinase plays a key role in decomposition and utilization of chitin as a raw material. This research indicates that *Myxococcus fulvus* UM01 strain is a novel myxobacteria strain and can produce large amounts of chitinase within a short time. The UMCda gene cloned into *E*. *coli DH5a* showed a promising effect as antifungal activity. In overall findings, the specific strain UM01 has endowed properties of bioconversation of waste chitin and other biological applications.

## Background

Chitin is the second most abundant natural biopolymer after cellulose. The chemical structure of chitin is similar to that of cellulose with 2-acetamido2-deoxy-b-d-glucose (NAG) monomers attached via β(1→ 4) linkages, with the chitin word derived from the Greek “chiton” which means coat of mail [1]. In 1828, French botanist Henri Braconnot noticed that polysaccharide took title chitin as he researched edible mushrooms [2]. Chitin (C_8_H_13_O_5_N)_n_ is related to a cellulose that is a long 2-acetamido-2-deoxy-β-d-glucose (NAG) monomer; the units are linked via β(1→4). Chitin is semitransparent, and it is found in the exoskeleton of crabs, lobsters, and insects, the cell walls of fungi, the radula of mollusks, and also in the internal structures of invertebrates. Chitin serves several purposes such as strengthening the internal and external structures. Chitin is the second most common natural polysaccharide (β-(1-4)-N-acetyl-d-glucosamine), with cellulose being the first in the polymer world [3]. Chitin found in α-chitin and β-chitin can be characterized by infrared and in solid-state NMR, together with X-ray diffraction [4]. The third allomorph form, γ-chitin, was characterized with different chemical properties [5], but detailed investigations show that it is just a variant of α-chitin [6].

Throughout years of research on this polymer, different methods have been developed for extraction. Most of the methods discussed in this literature are chemical methods used for the production of industrial chitin. The USA, Japan, Canada, China, Russia, Norway, and India largely reject the use of crustacean fishing for production [7]. Chitin extraction using strong acids and bases triggers serious consequences to process, such as low level of purity, generation of chemical effluents, and increased cost of production [8, 9]. The high quality of the final product, with affordable COP biological processes, is more attractive to various industries [10, 11]. The main industrial sources for biopolymer extraction are byproducts of the fishing industry, such as prawns, crabs, and lobster shells [12, 13].

Annual production of chitin and its derivatives from living oceanic organisms are estimated at 10^12^–10^14^ tons [14]. The cost of chitin is estimated at $220/kg on the world market [15]. In order to utilize the excess quantity of chitin, developing commercial procedures for extraction of competent polymer are needed and may reduce the harmful impacts it has on the environment, biodiversity, and human health. Chitin and its deacetylated form chitosan each are valuable economic commodities due to their elasticity of biological properties, such as crystallinity and strict insolubility. Chitin and its derivatives COS, chitosan, and glucosamine augments increase their application in the food, textile, medical, agriculture, and cosmetic industries. Chitinase bacteria are capable for hydrolyzing insoluble chitin to its oligo component and are found in various organism including bacteria, virus, fungi, insect, plant, and animal and play different physiological roles. Chitinase is an essential part of numerous bacterial species; they are well known specifically in the genera *Aeromonas*, *Serratia*, *Vibrio*, *Bacillus*, *Streptomyces*, and *Myxobacteria*. Our study aimed to report on screening, gene cloning, and antagonistic and biochemical representation of the novel strain UM01 from *Myxococcus fulvus*. Remarkably, the enzyme was discovered and is suitable against antifungal activity. To the best of our knowledge, this is the first research on chitinase from *M*. *fulvus*.

## Method

### Collection of samples and chitinolytic bacteria isolation

A total of 10 samples were collected from different areas of Jiangsu China. Four soil samples were collected form Lihu Lake (Wuxi) and Xihu Lake (Hangzhou), and the remaining 6 samples were collected from maize rhizophore and the fish market. For screening purposes, agar media containing colloidal chitin and colonies with a clear zone were used and considered for further study for chitinase, as these colonies specify chitinase-producing bacteria.

### Colloidal chitin preparation

For colloidal chitin, preparation powder chitin (case no.1398-61-4) was purchased from Sigma-Aldrich and prepared by slight modification in Hsu and with the Lockwood method [16]. Ten grams of powdered chitin was prepared by dissolving 300 mL conc. HCL gradually, acid was added to chitin with stirring, and mixture was allowed to stand at room temperature with alternating stirring until the chitin dissolved (2 h). By using 8 layers of cheesecloth (to remove large chitin chunks), the chitin-HCL solution was poured into a 2-l ice-cold (4 °C) D_2_H_2_O beaker and kept in the fridge overnight at (4 °C). After 24 h, there will be white cake-like chitin in the solution. Next, we have to centrifuge the white cake-like chitin solution at 18,000 rpm (F0485 Fix-Angle-Rotor) for 15 min. We then proceeded to wash it with D_2_H_2_O, at least 4 times until pH 5 is reached. Lastly, we loosened the colloidal by using 100 mL distill water to make w\w solution and stored it at 4 °C with an aluminum covering.

### Culture media preparation

For identification of bacteria, LB (Luria broth) is the most commonly used medium; for screening chitinase-producing bacteria, selective medium is used. Selective medium containing (g L^−1^) KH_2_PO_4,_ 14; K_2_HPO_4_, 6; (NH_4_)_2_SO_4_, 2; Na_3_C_6_H_5_O_7_, 1; MgSO_4_, 0.12; Agar, 15; with 1% (w/v) colloidal chitin at pH 7, were incubated at 30 to 37 °C. The clearance zone was formed by chitin hydrolysis and was recorded up to 8 days. Based on the size of clearance zone, two of the best samples were chosen for further study.

### Gram staining procedure

The standard gram staining procedure was performed for the characterization of isolate [17].

### Biochemical tests

Different chemical tests were performed to test the motility and oxidase. The nitrate reduction test and urease test were used for characterization [18].

### Enzyme assay

Colloidal chitin was used as the substrate for measuring the chitinase activity according to originally modified protocol [19]. Reaction mixture consists of 0.5 mL enzyme which was added to 0.5 mL colloidal chitin (1% w/v); it was then incubated at 45 °C for 1 h. The reaction was terminated at 90 °C for 5 min after incubation. The reaction mixture was then centrifuged at 20,000*g* (F0485 Fix-Angle-Rotor) for 5 min, and the supernatant was collected. The supernatant was boiled with 0.8 M potassium tetraborate of 100 μL quantity and boiled for 3 min, followed by the addition of 1 mL of dimethylamino benzaldehyde for 30 min at room temperature, and then incubated until it appeared to be a pink color. Absorbance was measured at 560 nm by using a spectrophotometer. A blank solution was used as a negative control. One chitinase enzyme unit was defined as a change in the absorbance of 0.01 min^–1^ [20].

### Molecular characterization of microorganism DNA sequencing

The gene coding 16S rRNA was sequenced to identify the genus of each strain. The genomic DNA of the selected strains were isolated by using the TIANamp Bacterial DNA kit, and amplification was performed by using the PCR master mix kit with initial heating step for 2 min at 95 °C followed by denaturation step 30 cycles of 95 °C for 30s, annealing for 60 s at 55 °C, and 2 min extension at 72 °C, and then followed by final extension at 72 °C for 7 min. Two universal primers FC 27 (forward) and RC1429 (reverse) were used for amplification. The amplified product was than purified by using DNA purification kit, the purified product was sent to Sangon Biotech Shanghai for sequencing, and 16S rRNA sequence was obtained. 16S rRNA sequence analysis showed that this strain belongs to genus *Myxococcus* and species *Myxococcus fulvus*.

### Phylogenetic analysis

The gene coding 16S rRNA partial sequence obtained from Sangon Biotech Shanghai (www.sangon.com) was compared to other bacterial sequences to search for their pairwise identities by using NCBI, BLAST (BLAST; http://www.ncbi.nlm.nih.gov/BLAST/), and online software. Multiples sequence alignments of highly similarity sequences were available at the data bank and were performed by using MEGAX (Version 10.1.5). By using the neighbor-joining method (NJ), phylogenetic analysis was carried out with the MEGAX (Version 10.1.5) [21]. Partial gene coding 16S rRNA sequence was then submitted to NCBI Gene bank with the name UM01 under the accession number MN811202.1 [22].

### Determination of different substrates and effect on enzyme production

To determine the best substrate for chitinase production, various substrates such as (1%) colloidal chitin, powdery chitin, crab shell, and chitosan were used. Final 20 mg/mL substrate concentration was used in the final reaction mixture and the increase reductively was determined [23].

### Effect of temperature and pH on enzyme production

The effect of temperature for optimum enzyme production was determined at 20 to 55 °C by using colloidal chitin as a substrate, and the pH value effect on enzyme production was examined by varying pHs from pH 3.5 to pH 9 in culture medium at optimized temperatures and incubation period. Tris-*HCL* buffer (pH 6.5 to 9) and acetate buffer (pH 3.5 to 6.0) were used for this purpose.

### Chitinase gene amplification

Five starter primers were used for amplification of chitinase gene from UM01 strain:

**BBGA (**F): ATGAGCACAAATAACATTATTAATGC, **BBGA** (R): TTAGGCGATGAGCTGCC

**BBGB** (F): ATGCAGCTTCCAGGATTCC, **BBGB** (R): TTACGGGTAGGTGTCCAGGT

**BBGC** (F): ATGCAGAGGTCCCTCGC, **BBGC** (R): TTAGCGCACG AACTCCC

**BBGD** (F): ATGTCTGGCAATTTTGTTTCAC, **BBGD** (R): TTAGCAGCCCAGGTTGC

**BBGE** (F): ATGGCCATGGCCGTG, **BBGE** (R): TTAGGGCTGGAAGGCTTCC

All these primers were designed by searching the database (NCBI) to find the closest relative sequence. By putting all these sequences in FASTA format in a text document, we were able to do multiple alignments using Clustal Omega. For amplification of the chitinase gene, genomic DNA from strain UM01 was used by using TIANamp bacterial DNA kit and amplification was performed by using PCR master mix kit by following standard PCR protocol. The obtained PCR product was analyzed by gel electrophoresis and an amplified purified product was sent to Sangon Biotech Shanghai for sequencing.

### Cloning of (UMCda) chitinase gene

The coding region of the chitinase gene (UMCda) was amplified by PCR with the flanking restriction enzyme by using forward primer **BBG71F:** CCGGAATTCATGAACAAAACTTCCCGTAC and reverse primer **BBG7R:** CCCAAGCTTTTATTTGCTCAGGTTGACGT, and PCR products were purified by using PCR purification kit (Thermo fisher). By using the restriction enzyme EcoR1 and HIND111, the PCR product were digested and then ligated into pET-28 plasmid vector, which was digested with the same restriction enzyme. By using the heat shock method, the recombinant vector transformed into *E*. *coli DH5a*. The recombinant *E*. *coli DH5a* strain was grown in LB medium containing 100 mg/mL kanamycin at 37 °C in a rotatory shaker 180 rpm. When the optical density (OD = 600) of culture media has reached up to 0.5, expression of recombinant protein was induced by IPTG 0.5 mM and culture was grown at 37 °C at 12 h.

### Purification of recombinant chitinase and chitinase assay

The reaction mixture containing 0.1 mL enzyme solution and 0.1 mL (1%) colloidal chitin which was incubated at 45 °C for 30 min, and the reducing sugar was determined [23]. The one unit of enzyme activity is defined 1 μmol of GlcNAc obtained from enzyme hydrolysis during 1 min using 1 mL of enzyme.

For purification of recombinant chitinase, 1 mL of overnight culture was induced with IPTG; the cell was then harvested by using centrifugation at 4 °C at 10,000 rpm and re-suspended again in 1 mL PBS buffer then centrifuged again 12,000 rpm for 1 min, followed by a second wash with 55 μL PBS buffer. The cell was disrupted by using sonication and again centrifuged at 4 °C at 10,000 rpm for 30 min to obtain suspension. Purification of the enzyme was done by using NI-NTA affinity chromatography and imidazole used as an eluent. Column chromatography purified chitinase enzyme was loaded to DEAE-cellulose A52 (2.6 × 20 cm) column. A different salinity of tris 0.05 M HCL and pH 7 was applied. Elution step was preceded in a stepwise gradient of NaCl (0–1.0%) at the flow rate of 0.5 mL/min. The selected fraction was applied for Sephadex G-100 column (1.6 × 30). Purified column was eluted with tris 0.05 M HCL and at pH 7 with the rate of 0.4 mL/min. The fraction of each 3 mL was collected and chitinase activity with protein content of each fraction was determined accordingly [24]. The purified beak was chosen for determining the properties of chitinase enzyme. SDS-PAGE using 12% gel in Tris-glycine buffer pH 8.3 [25] was done to determine enzyme homogeneity. Chitinase activity was measured by using a modified method [26].

### Antifungal activity of chitinase gene

To evaluate the antifungal activity of the recombinant chitinase gene, it was measured against *Trichoderma reesei* IFO 31329 using a chitinase plate assay by using a slightly modified method of the previous study [27]. Recombinant strain was streaked at the center of the agar plates containing 1:1 (v/v) ratio of potato dextrose agar (PDA) and yeast extract mannitol) (YEM) with the addition of 0.5% colloidal chitin and was incubated at 37 °C for 24 h for bacterial growth. Next, we inoculated each bacterial (recombinant) colony and the mentioned strain in a straight line. After a 24-h incubation period at 37 °C, a mycelial lump of *T*. *reesei* was placed on the center of the plate. One plate with bacterial inoculation was used as a control. Both plates were incubated at 37 °C for 3 days, and the antifungal activity of recombinant was evaluated by visual examination.

## Results

A total of two chitinolytic bacteria were identified by screening the 10 soil samples that were collected from different regions in China. Based on colloidal degradation and the clear zone, two colonies were identified as the best; both of them were used for secondary screening by using broth media and enzyme assays. Based on the chitinase production, one isolate was used for further study.

### Identification and molecular phylogeny of bacteria

Identification of the novel isolated bacteria strain UM01 was done by both the catabolic and molecular methods. The strain identified as *M*. *fulvus* is a typical species of the genus *Myxococcus* in the family *Myxococcaceae*. Colonies on the agar plate were raised, were smooth, and were a light-yellow color which turned light pink with time. Classification and morphological properties of strain UM01 are shown in (Table 1).

**Table 1:** Classification and morphological properties of *Myxococcus fulvus* UM01 strain

Morphological characteristics	
Gram staining	Negative
Form	Rod
Motility	Positive
Spore	Positive
Habitat	Soil
Carbon source	Macromolecules
Oxygen requirement	Aerobic
Pathogenicity	Non-pathogen
Biosafety level	1
Geographical location	China
**Classification**	
Phylum	Proteobacteria
Class	Deltaproteobacteria
Order	Myxococcales
Family	Myxococcaceae
Genus	*Myxococcus*
Species	*Myxococcus fulvus*
Strain	UM01

### Gene coding 16S rRNA analysis

**Fig. 1:**
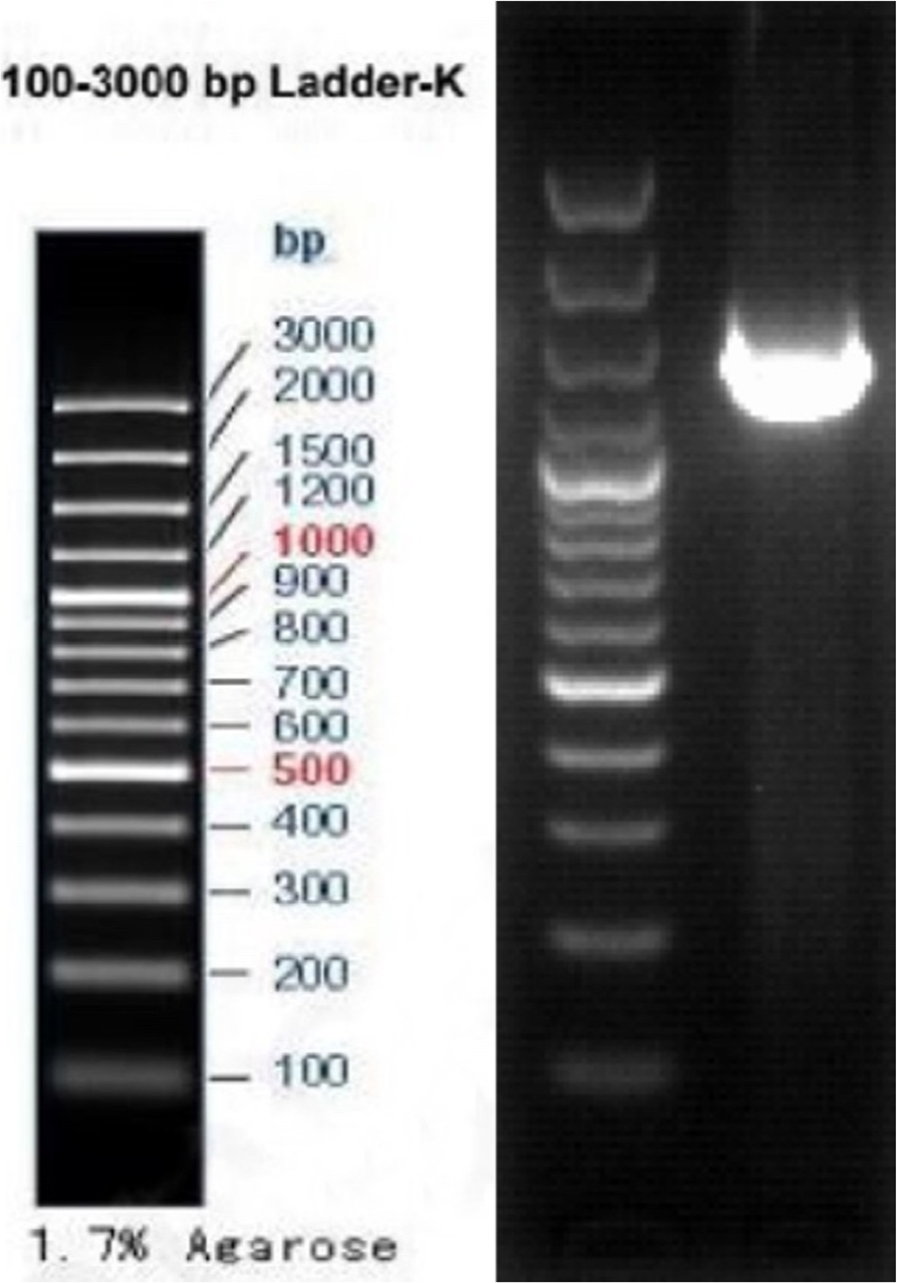
Gene 16S rRNA of *M*. *fulvus* UM01 strain

A 1476 bp of 16S rRNA gene was sequenced from strain UM01. Gel pictures of the amplified product shown in Fig. 1 were submitted to NCBI under accession no. MN811202.1. Strain UM01 shows maximum homology with strain *Myxococcus fulvus* strain 0195-1 and *Myxococcus* sp. SDU-1.

### Phylogenetic tree analysis

**Fig. 2:**
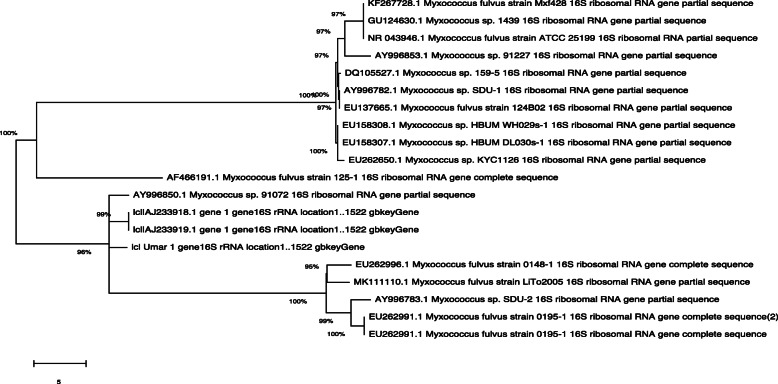
Phylogenetic analysis of *M*. *fulvus* UM01 strain mentioned in this tree with the name ICI Umar 1 gene 16S rRNA showing its similarity with AY996850.1 *Myxococcus* sp. 91072

Phylogenetic analysis was performed by using MEGAX (Version 10.1.5). Only the highest sequence of homology score was used after NCBI Blast was considered for constructed phylogenetic tree shown in Fig. 2.

### Effect of pH on chitinase production

**Fig. 3:**
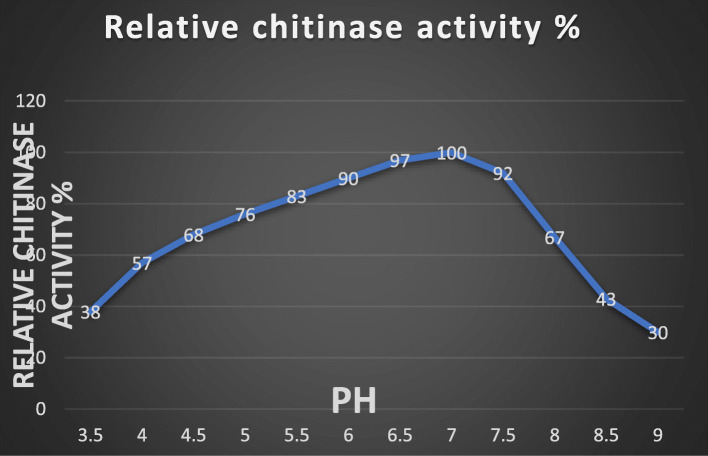
Effect of pH on chitinase production by UM01

To evaluate enzyme activity and stability, bacteria culture was grown at different pH ranges such as pH 3.5 to pH 9. The optimum pH of UM01 chitinase production was at pH 7, and its activity was stable at pH 5.5 to pH 8 as shown in Fig. 3. The above results indicated that the pH of the media individually did not only help chitinase production but it also performed a key role in cell growth. Like *Myxococcus fulvus* 124B02, the study previously also reported optimum pH 6 to pH 7.6 for bacterial growth [18].

### Effect of temperature on chitinase production

**Fig. 4:**
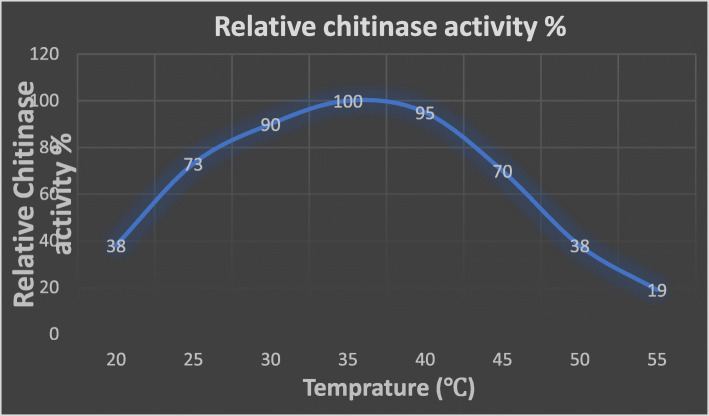
Effect of temperature on chitinase production by UM01

Temperature is a key factor for various biological processes for bacterial growth and enzyme production, which also is affected by a change in incubation temperature. To measure enzyme activity and stability, UM01 was grown at a different incubation temperature which ranges from 20 to 55 °C. Chitinase production was highest at 35 °C. With increased temperatures of 40 to 55 °C, the enzyme activity started decreasing as shown in Fig. 4; *Myxococcus fulvus 124B02* and *Myxococcus fulvus* KYC4048 both show optimum growth at 32 °C [18, 28].

### Effect of substrate on chitinase production

**Fig. 5:**
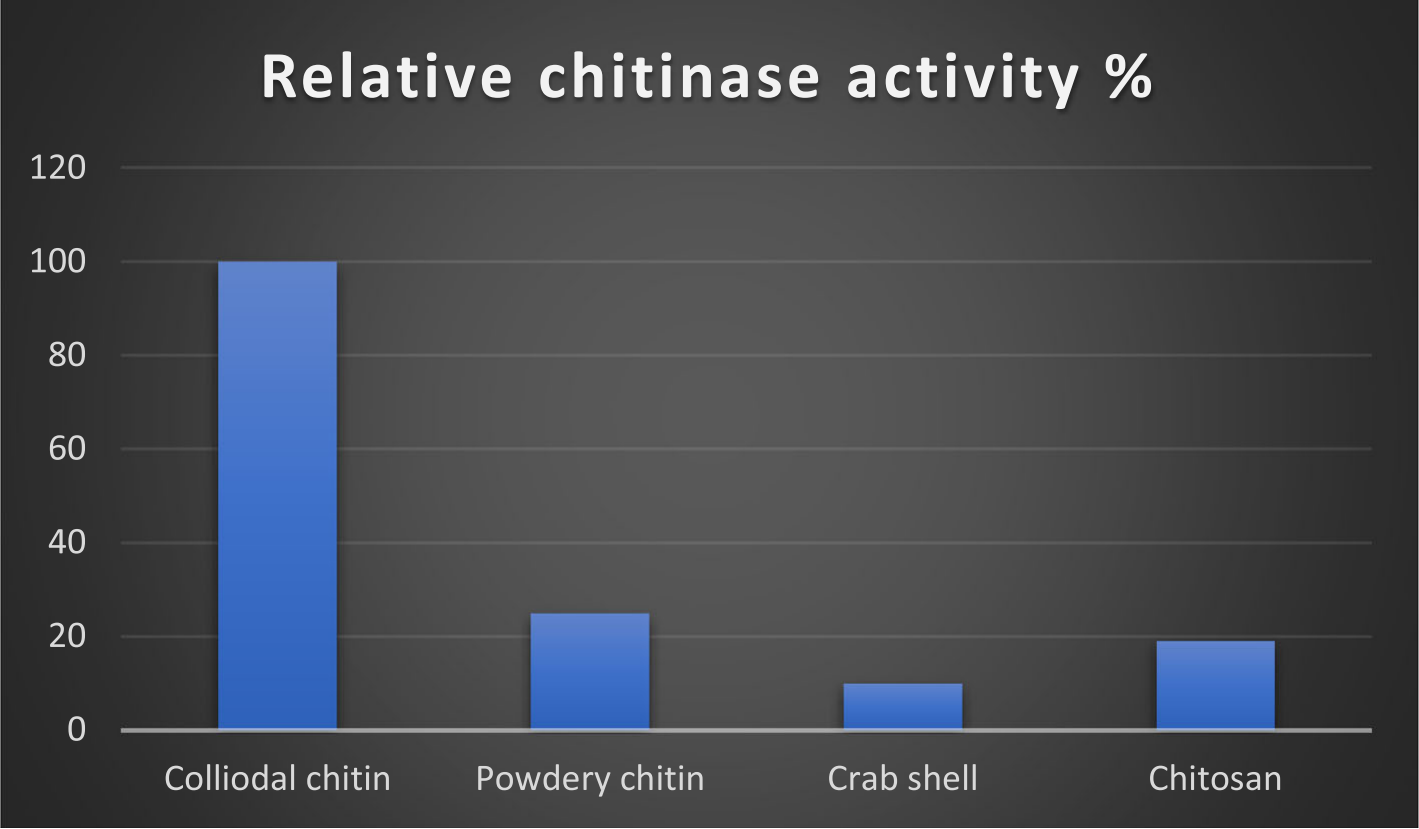
Effect of different substrates on chitinase production by UM01

To specifically measure the best substrate in chitinase production, the strain UM01 was used in different substrates such as colloidal chitin, powdery chitin, crab shell, and chitosan. Among all, these colloidal chitins were found to be the best for chitinase production, and powdery chitin and crab shell had lower activity as shown in Fig. 5. The previous study also proved that *Eisenia fetida* shows great chitinase activity by using colloidal chitin as a substrate [29].

### Sequence analysis of UM01 chitinase-producing gene

**Fig. 6:**
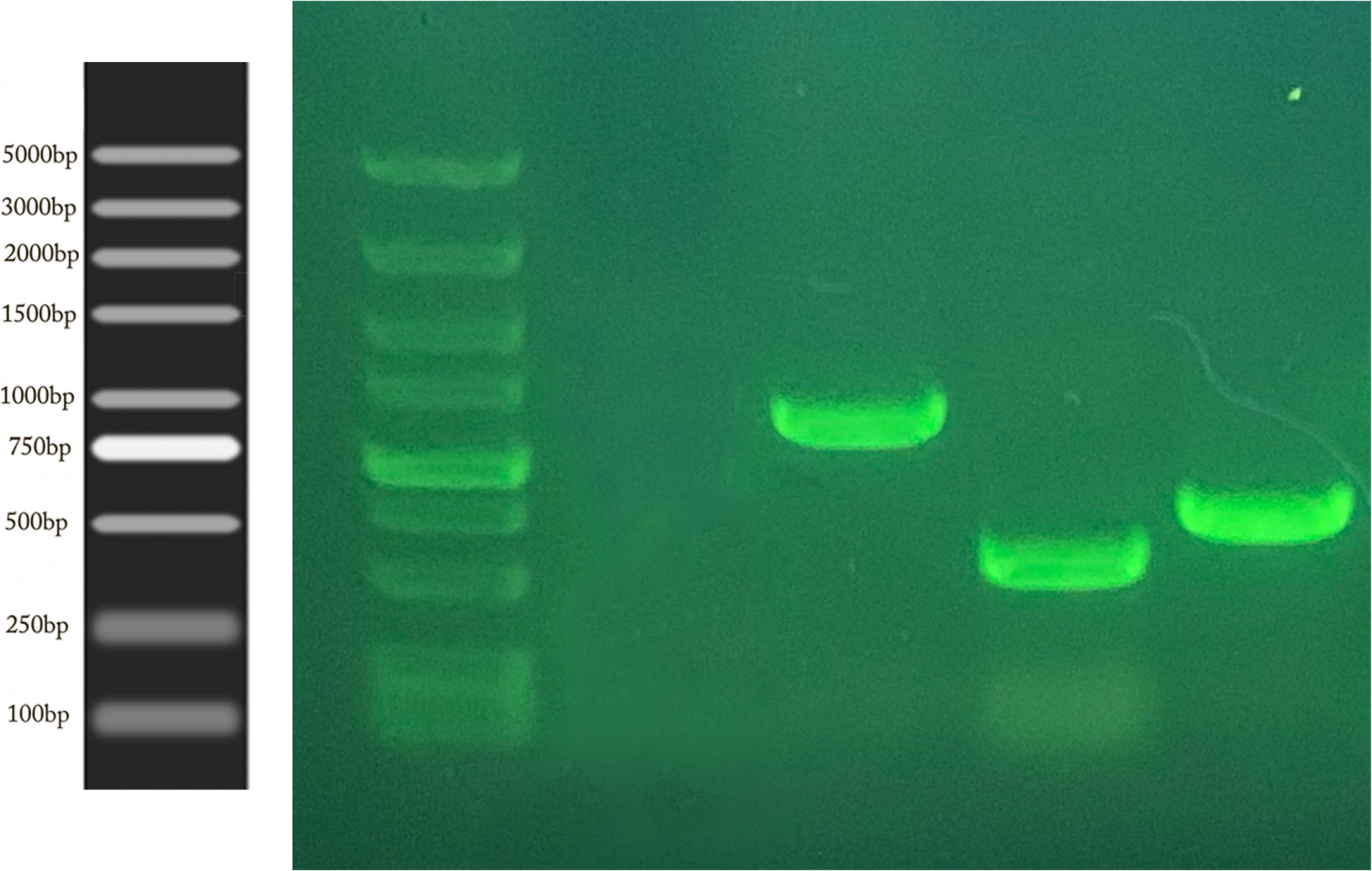
By using degenerative primer amplification of 3 chitinase-producing genes from *M*. *fulvus* UM01 strain

By using the degenerative primer, 3 chitinase-coding genes were identified after PCR amplification gel electrophoresis was run to analyze these genes as shown in Fig. 6. All 3 genes were sequenced by Sangon Biotech Shanghai, for sequence analysis. Chitinase18 (glycoside hydrolase family 18) is 1024 bp long. Sequence comparison was done by using the online software NCBI BLAST (http://www.ncbi.nlm.nih.gov/BLAST/) alignment with another glycoside hydrolase gene derived from *Serratia Marcescens* strain WVU-005, *Serratia Marcescens* strain UMH12, and *Serratia Marcescens* subsp. *Marcescens Db11* obtained with the sequence identity of 94.93% and the *E* value = 0. UMCBP (chitin-binding protein) 592 bp long gene by sequence comparison with online tool NCBI BLAST and alignment with another CBP gene from *Serratia Marcescens* strain E28, *Serratia Marcescens* strain CAV1761, and *Serratia Marcescens* strain S2I7 was obtained with the sequence identity of 97.4 to 97.0% and the *E* value = 0. UMCda (chitin disaccharide deacetylase) 749 bp long gene after NCBI blast alignment with the other Cda genes derived from *Serratia Marcescens* isolate GN26, *Serratia Marcescens* SMB2099, and *Serratia Marcescens strain* C110 was obtained with sequence identity 95.5 to 96.3% and the *E* value = 0.

### Cloning and purification of UMCda gene

**Fig. 7:**
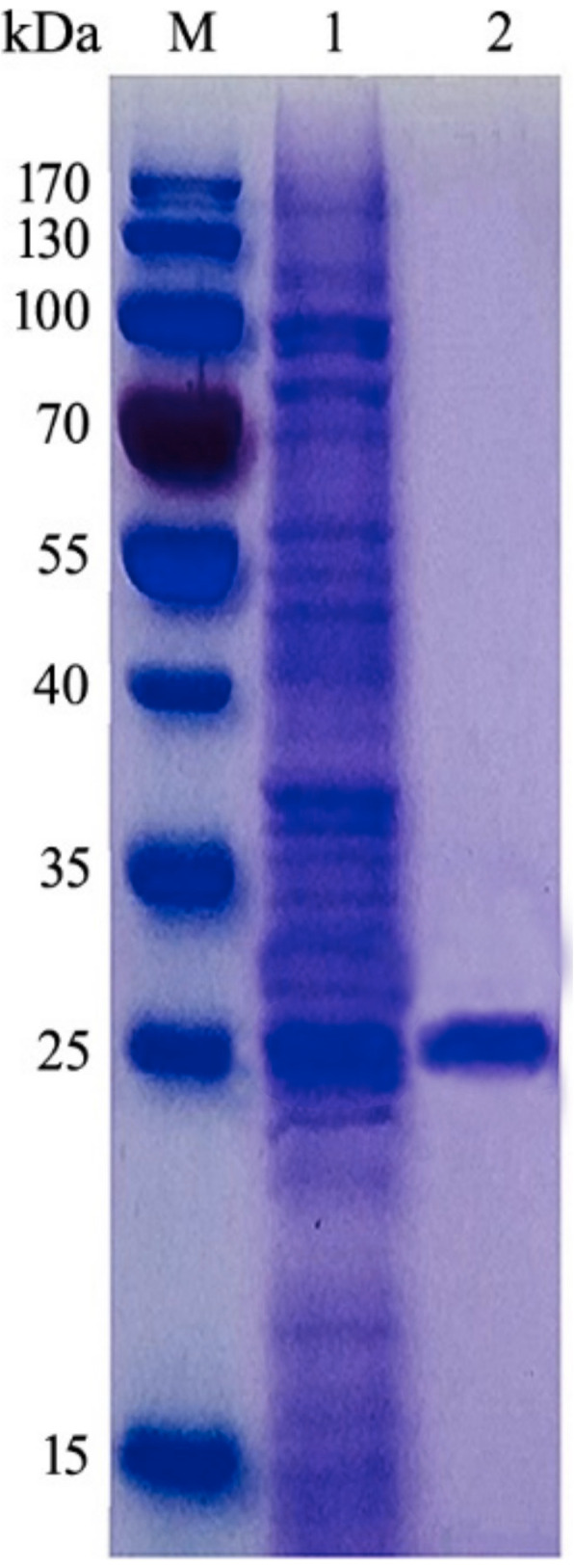
SDS-PAGE analysis of purified UMCda from *M*. *fulvus* UM01 expressed in *E*. *coli*. M, molecular weight marker; 1, crude protein extract; 2, purified chitinase

**Fig. 8:**
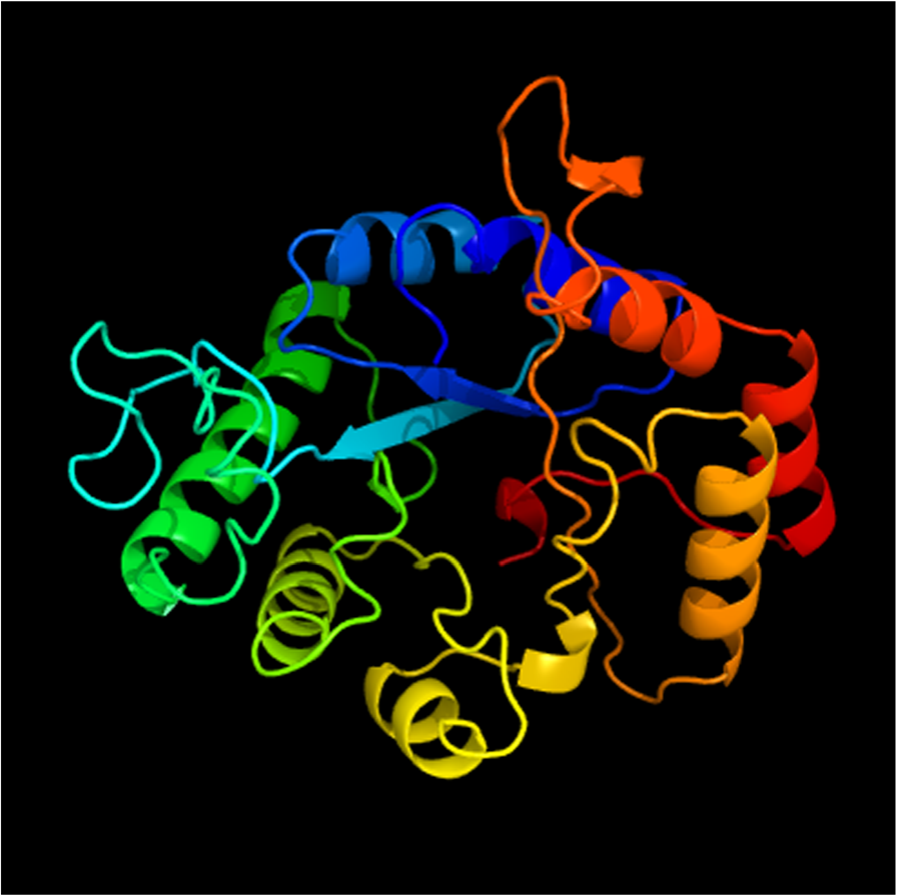
3D structure prediction of UMCda protein

**Fig. 9:**
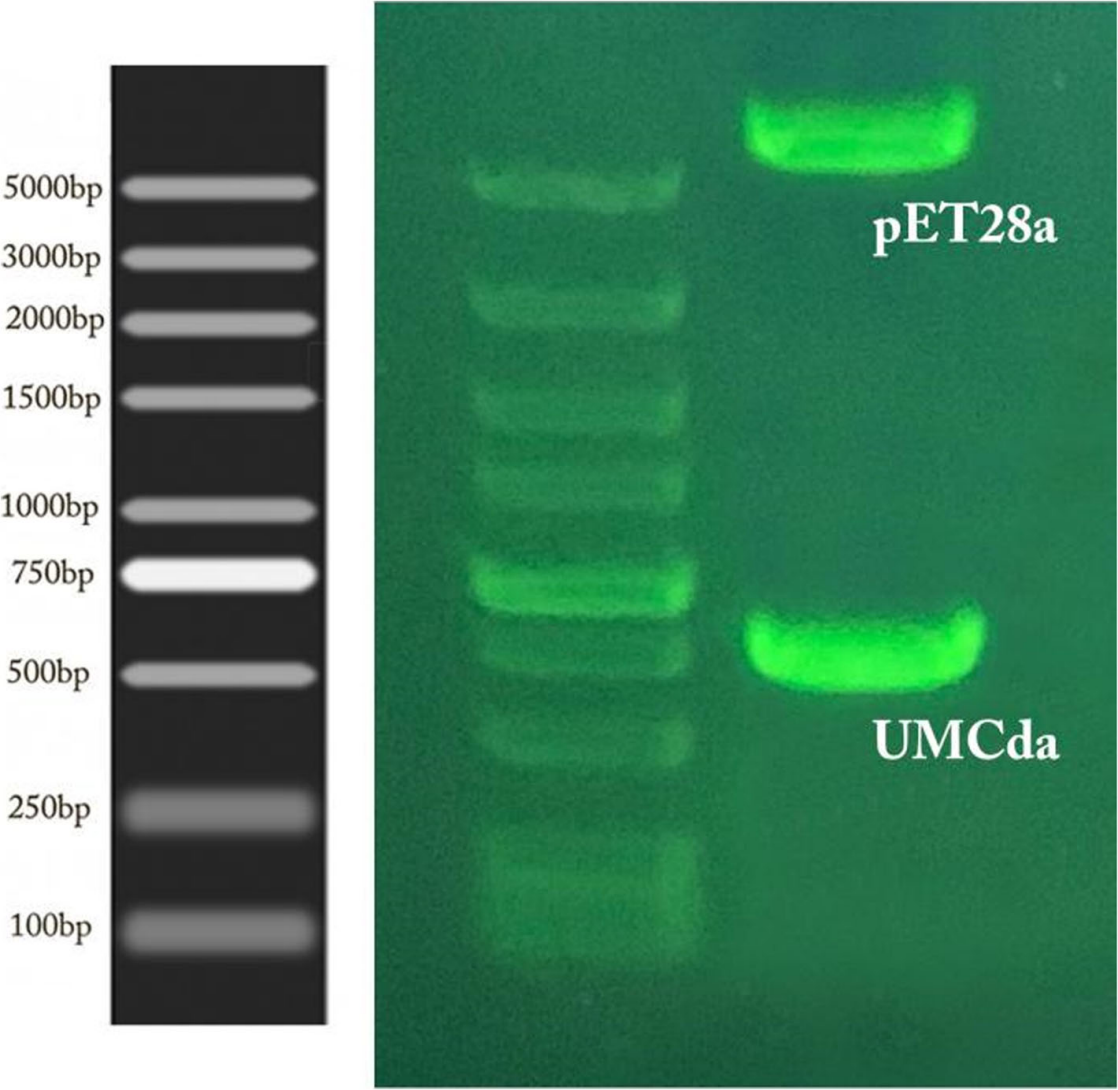
Gel electrophoresis analysis of pET-28a vector and UMCda gene after successful transformation

UMCda gene CDS was submitted to Banklt under accession no. MT249254.1 [30] and was overexpressed into *E*. *coli DH5a* by using PET-28a vector, with and without signal peptide exhibited; there was no alternation on protein expression and enzyme activity. Gel electrophoresis analysis of pET-28a vector and UMCda gene after a restriction cut with the same enzyme is shown in Fig. 9. About 60.2 mg of recombinant UMCda protein was obtained from 1 l of culture by using Ni-affinity chromatography. The molecular weight of UMCda is approximately 26.99 kDa measured by using SDS-PAGE analysis as shown in Fig. 7. For structure prediction analysis, online tool Phyre2 was used for protein 3D structure. Protein UMCda shows 100% confidence and 98% coverage and i.d % 37 with fold 7-stranded beta/alpha barrel, superfamily glycoside hydrolase/deacetylase, and family YdjC-like9 (Fig. 8).

### Antifungal activity of UmCda gene

**Fig. 10:**
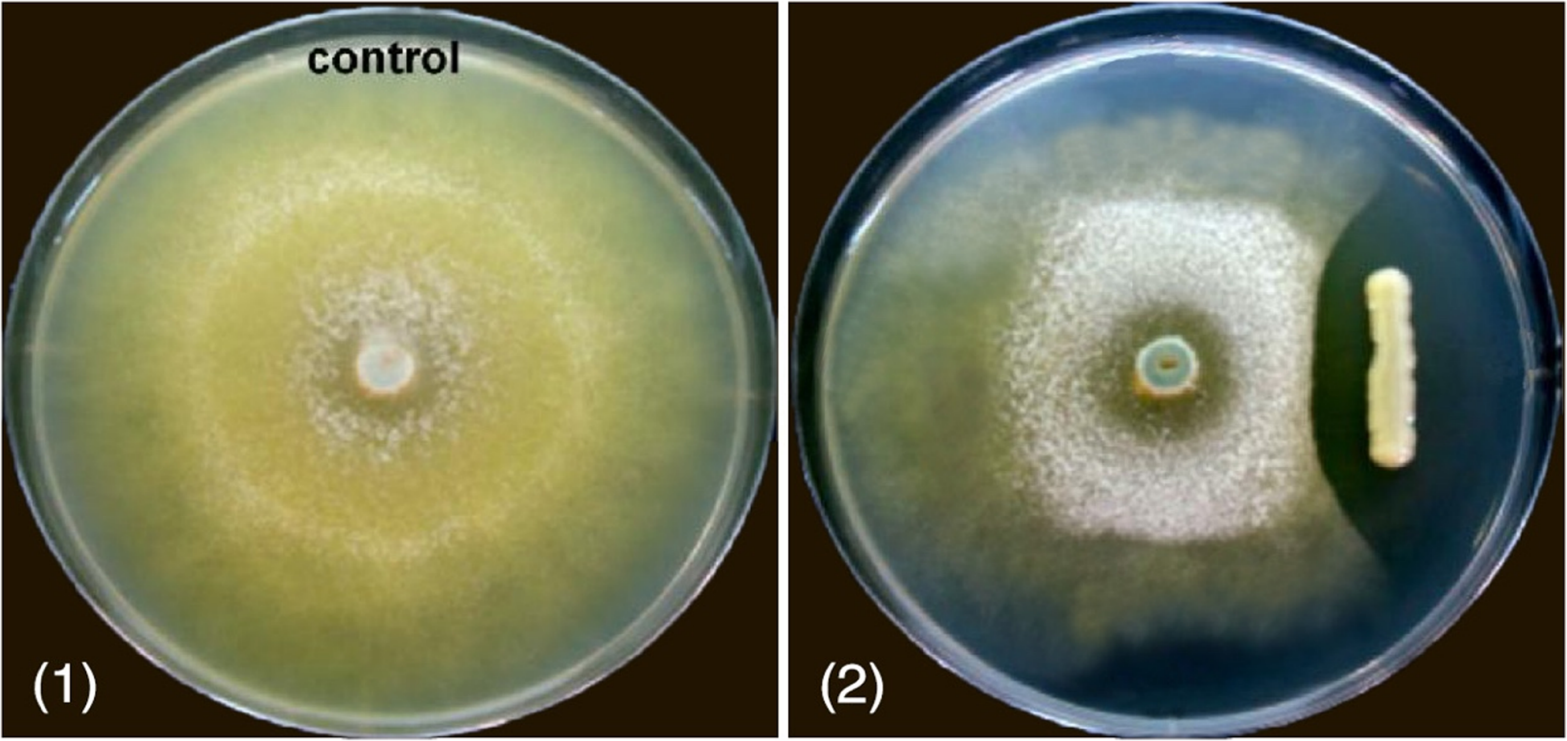
Antagonistic properties of UMCda against hyphal growth of *T*. *reesei* by dual culture assay. **1** Plate used as a control. **2** Antifungal activity of UMCda

The UM01 strain was further studied against the antifungal activity (Fig. 9). The selected gene UMCda was cloned, and the antifungal activity of UMCda was examined against *T*. *reesei* by dual-culture assay as shown in Fig. 10. The recombinant UMCda gene shows a strong antagonistic activity. Antagonistic activity was also reported *by Cellulosimicrobium cellulans* 191 [31] and *Myxococcus* sp. KYC 1136, 1126, and 2001 were tested for antifungal activity in vitro [32]. Hyphal inflation was followed by distortion [33], and cell wall secretion of the chitin oligosaccharides and cytoplasmic outflow were detected by fungi after chitinase was affected [34].

## Discussion

Chitin is the second most abundant polysaccharide, after cellulose, in the marine ecosystem. Chitin naturally exists in numerous sources such as insects (ladybugs, silkworms, wax worms, and butterflies), mollusks (oyster shells and squid pens), crustaceans (shrimp, lobster, and crab), and fungi (*Mucor rouxii*, *Aspergillus niger*, *Penicillium chrysogenum*, and *Lactarius vellereus*) [35]. Myxobacteria are recognized because of their large size genome beyond 9 Mb and their social behaviors. *Myxococcus fulvus* belongs to the genus *Myxococcus* in the Myxococcaceae family. In this study, chitinase UMCda was expressed by *E*. *coli DH5a* and showed the highest activity on colloidal chitin than the other substance. Meanwhile, the main product of chitin is GlcNAc_2_; other oligosaccharides are not detected, which provide convenience for the purification of product. Recently, chitin oligosaccharides gained lot of research attention worldwide because of their potential use in biomedical, agriculture, and food industries. *Myxococcus* species is well known due to its potential biocontrol agent and chitinase production; various reports have specified chitinase production from *Myxococcus xanthus* [36]. This research reports first time the chitinase gene production from *Myxococcus fulvus* screened from soil. Chitinase from *Myxococcus fulvus* exhibited maximum activity on colloidal chitin. The fact is that the individual chitinase are often different in their optimum pH and optimum temperature even from same species. The purified UM01 shows more optimum compared with that earlier studied from *Myxococcus fulvus* 32 °C [28] and from *Serratia marcescens* 45 °C. The optimal pH value of chitinase enzyme UM01 determined pH 7 as its lower value compared with *Myxococcus xanthus* with pH 7.5 [37]. Single sharp band at 26.9 kDa was determined as the molecular weight of the one purified, which is approximately close to that of chitinase produced from *Monacrosporium thaumasium* (30 kDa), *Bacillus cereus 108* (30 kDa), and *Bacillus licheniformis strain jS* (22 kDa) [37–39]. Our study showed that *Myxococcus fulvus UM01* produced antifungal chitinase. The synthesis of chitinolytic enzyme depends on many factors, including composition of medium and the chitin source. The antifungal activity of *Myxococcus fulvus UM01* was examined using its cells and crude. Although the crude proteins distinctly inhibited the hyphal growth of *T*. *reesei*, the cells did not suppress mycelial extension. The antifungal activity of crude proteins could be due to other components such as antifungal proteins and/or antifungal antibiotics other than chitinases that remained in the crude protein fraction after dialysis. However, such antifungal proteins and antifungal antibiotics have not been reported yet from this bacterium. In addition, our observations revealed that this isolate grew poorly and slowly on solid media such as YEM, synthetic medium, tryptone soya broth, nutrient broth, and LB. This strain exhibited better growth on media containing glucose, colloidal chitin, or glycol chitin, but the growth of the strain was still slower than that of the other isolates.

## Conclusion

To our knowledge, this is the earliest study to report gene cloning and identification of the chitinase gene in *Myxococcus fulvus*. Chitinase plays a key role in decomposition and utilization of chitin as a raw material. This research indicates that the *Myxococcus fulvus* UM01 strain is a novel *myxobacteria* strain and can produce large amounts of chitinase within a short time. The UMCda gene cloned into *E*. *coli* DH5a showed a promising effect with antifungal activity. In overall findings, the specific strain UM01 has endowed properties of bioconversation of waste chitin and other biological applications. There is a need for further study of the enzyme action and 3D protein structure of UMCda protein. Chitin deacetylase can hydrolyze acetamide, a group of chitin, and produce chitosan which can be used for biomedical, pharmaceutical, food, and agriculture industries.

## Data Availability

16S rRNA gene of strain UM01 submitted to NCBI under accession no. MN811202.1 UMCda gene CDS submitted to Banklt under accession no. MT24925.

## Abbreviations

COP: Cost of production
COS: Chitosan oligosaccharides
CBP: Chitin-binding protein
PCR: Polymerase chain reaction
